# Does Long-Term Night Shift Work Cause Dry Eye in Hospital Nurses?

**DOI:** 10.18502/jovr.v18i4.14543

**Published:** 2023-11-30

**Authors:** Aria Bouyeh, Hassan Hashemi, Yousef Alizadeh, Ebrahim Jafarzadehpur, Ali Mirzajani, Hadi Ostadimoghaddam, Abbasali Yekta, Abolfazl Jafarzadehpour, Arghavan Zarei, Mehdi Khabazkhoob

**Affiliations:** ^1^Noor Research Center for Ophthalmic Epidemiology, Noor Eye Hospital, Tehran, Iran; ^2^Noor Ophthalmology Research Center, Noor Eye Hospital, Tehran, Iran; ^3^Eye Research Center, Guilan University of Medical Sciences, Rasht, Iran; ^4^Rehabilitation Research Center, Department of Optometry, School of Rehabilitation Sciences, Iran University of Medical Sciences, Tehran, Iran; ^5^Refractive Errors Research Center, Mashhad University of Medical Sciences, Mashhad, Iran; ^6^Department of Basic Sciences, School of Nursing and Midwifery, Shahid Beheshti University of Medical Sciences, Tehran, Iran

**Keywords:** Night Shift, Dry Eye, Tear Break-Up Time, Ocular Surface Disease Index, Nurses

## Abstract

**Purpose:**

To determine the long-term effects of night shift work on dry eye in hospital nurses.

**Methods:**

Each participant was evaluated four times, including at the beginning of the day shift (8 am), at the end of the day shift (2 pm), at the beginning of the night shift (8 pm), and at the end of the night shift (8 am), using the tear break-up time (TBUT) test and ocular surface disease index (OSDI) questionnaire.

**Results:**

The results showed significant differences in the TBUT and OSDI between the end of the day shift (2 pm) (10.26, 16.61) and the end of the night shift (8 am) (6.89, 38.59) relative to each other and relative to the beginning of the day and night shifts. As for the correlation between TBUT and OSDI, a significant correlation was found at all measurement times (correlation coefficient: - 0.478, - 0.707, - 0.556, and - 0.365, respectively) (*p* < 0.05).

**Conclusion:**

The results showed that the severity of dry eye increased after the night shift with variation over a 24-hr period. Moreover, a significant correlation was observed between TBUT and OSDI results at the beginning and at the end of the day and night shifts.

##  INTRODUCTION

The human sleep-wake cycle has a circadian rhythm that changes in different conditions during a 24-hr period.^[1]^ Regulation of the biological clock is related to ambient light changes, which regulate the physiology of peripheral organs through hormonal and neuronal signals.^[2]^ Sleep and circadian functions are essential for health. Sleep disorders are associated with changes in the autonomic and endocrine systems including hypertension, decreased parasympathetic tone, and increased secretion of stress hormones such as norepinephrine and cortisol.^[3]^ Physiological tear secretion is regulated by the cholinergic fibers of the parasympathetic nervous system, sympathetic stimulation, and hormonal factors. Stimulation of the sympathetic and parasympathetic nerves results in the release of neurotransmitters that control the secretion of proteins and electrolytes and the aqueous portion of the lacrimal gland.^[4,5]^ Therefore, sleep disorders may affect the tear film and ocular surface. Some studies have found that sleep disturbances affect the lacrimal system function resulting in tear hyperosmolarity, reduced tear break-up time, and decreased tear secretion.^[6,7]^

In 2003, Begley et al found that most of the dry eye symptoms increased in the evening as compared to the morning in patients with dry eye, while no significant difference was observed in most symptoms between the evening and the morning in the non-dry eye group.^[8]^

Night shift work is an inevitable part of the nursing profession.^[9]^ An important concern for night shift workers is the impairment of their normal circadian rhythm because they are exposed to light signals at work during night hours which can affect their job performance. It should be noted that the majority of the previous studies were conducted on people without a history of night shift work. The current study aims to investigate the signs and symptoms of dry eye at different times throughout the day in nurses with a relatively long history of night shift work.

**Table 1:** Frequency of the collected data in four measurement times.


orangeShift	orangeDry eye classification according to OSDI								orangeTBUT in seconds			
	<@orangeNormal (0–12)		<@orangeMild dry eye (13–22)		<@orangeModerate dry eye (23–32)		<@orangeSevere dry eye (33–100)		<@orangeDry eye (TBUT < 10 sec)		<@orangeNormal (TBUT ≥ 10 sec)	
	orangeN	orange%	orangeN	orange%	orangeN	orange%	orangeN	orange%	orangeN	orange%	orangeN	orange%
Beginning of Day shift (8 am)	4	10%	10	25%	11	27.5%	15	37.5%	28	70%	12	30%
End of Day shift (2 pm)	12	30%	16	40%	12	30%	0	0%	24	60%	16	40%
Beginning of night shift (8 pm)	3	7.5%	14	35%	6	15%	17	42.5%	27	67.5%	13	32.5%
End of Night shift (8 am)	1	2.5%	4	10%	8	20%	27	67.5%	39	97.5%	1	2.5%
TOTAL	20	12.5%	44	27.5%	37	23.1%	59	36.9%	118	73.8%	42	26.2%
	
	
white<bcol>13</ecol>OSDI, ocular surface disease index; TBUT, tear break-up time; sec, seconds

**Table 2:** Comparison of mean tear break up time and ocular surface disease index values between different measurement times.


orange**Shift**	orange**OSDI mean (S.D.)**	**orangeTBUT mean (S.D.) in sec**
Beginning of day shift (8 am)	26.82 ${}^{b}$ (9.76)	8.91 ${}^{b}$ (2.51)
End of day shift (2 pm)	16.61 ${}^{c}$ (7.2)	10.26 ${}^{a}$ (2.93)
Beginning of night shift (8 pm)	26.4 ${}^{b}$ (10.43)	9.12 ${}^{b}$ (3.33)
End of night shift (8 am)	38.59 ${}^{a}$ (12.85)	6.89 ${}^{c}$ (1.89)
F	253.1	28.56
Sig	< 0.001	< 0.001
	
	
white<bcol>3</ecol>- Similar letters in each column indicate a lack of significant difference at the level of *P* < 0.05. OSDI, ocular surface disease index;TBUT, tear break-up time; sec, seconds; S.D., standard deviation

**Table 3:** Comparison of mean tear break up time and ocular surface disease index values between the beginning and end of day and night shifts.


orange**Morning shift**				orange**Night shift**		
Beginning of day shift (8 am)		End of day shift (2 pm)	sig	Beginning of night shift (8 pm)	End of night shift (2 pm)	sig
TBUT	8.91 (2.5)	10.26 (2.93)	< 0.001	9.12 (3.33)	6.89 (1.89)	< 0.001
OSDI	26.82 (9.76)	16.61 (7.2)	< 0.001	26.4 (10.43)	38.59 (12.85)	< 0.001
	
	
white<bcol>7</ecol>OSDI, ocular surface disease index;TBUT, tear break-up time

**Table 4:** Correlation between tear break up time and ocular surface disease index tests in four measurement times.


orange**Shift**	orange**Correlation coefficient**	orange**P value**
Beginning of day shift (8 am)	- 0.478 ${}^{**}$	0.002
End of day shift (2 pm)	- 0.707 ${}^{**}$	< 0.001
Beginning of night shift (8 pm)	- 0.556 ${}^{**}$	< 0.001
End of night shift (8 am)	- 0.365 ${}^{*}$	0.021
	
	
white<bcol>3</ecol>- * indicates significance at the level of *P* < 0.05, and ** indicates significance at the level of *P* < 0.01.

**Figure 1:**
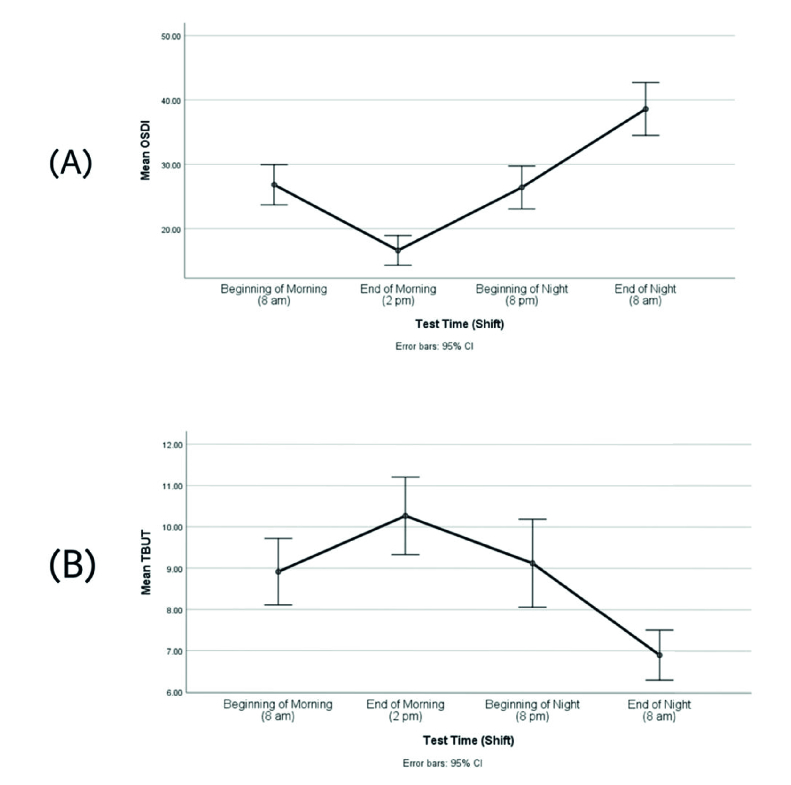
Comparison of mean ocular surface disease index scores between four measurement times (A) andcomparison of mean tear break-up time values (in seconds) between four measurement times (B).

##  METHODS

This cross-sectional study was performed in Amir Al-Momenin Hospital, Rasht, Guilan Province, from January to February 2020. The nurses who met the inclusion criteria were selected. Before performing diagnostic tests, the objectives of the study were explained to the subjects and informed consent was obtained from all of them. Forty female nurses who met the inclusion criteria participated in this study. The inclusion criteria were a history of night shift work in the last year for at least six nights per month; a lack of ocular pathologies such as eyelid problems, conjunctivitis, significant meibomian gland dysfunction (MGD), and lacrimal system diseases; no history of ocular surgery; not using systemic drugs causing dry eye such as antihistamines, anticholinergics, etc.; not using eye drops; not wearing contact lenses; lack of problems such as depression, mental disorders, and smoking; lack of eye-related systemic diseases such as rheumatoid arthritis, lupus erythematosus, and Sjogren's syndrome; and no history of gastrointestinal diseases or diseases associated with vitamin A disorders at the time of the study. In the exclusion criteria were as follows, the nurses who were unwilling to participate in the study, who were unable to undergo the TBUT during the examination, did not complete their night shift, did not have a good night's sleep during the night when they were off-duty but were scheduled to be examined in the following morning. Written informed consent was obtained from all participants before the examination. Then, after preliminary examinations, the nurses completed a demographic and work experience questionnaire as well as the OSDI questionnaire.^[10]^ Each participant completed the OSDI questionnaire at the beginning of the day shift (8 am), at the end of the day shift (2 pm), at the beginning of the night shift (8 pm), and at the end of the night shift (8 am). Moreover, the TBUT test^[11]^ was done immediately after completing the OSDI at the above times using the Haag Streit BQ900slit lamp and the results were recorded.

### Statistical Analysis

The SPSS version 19 was used for data analysis. The data was first cleaned and then analyzed using descriptive methods like frequency distribution tables and descriptive statistics. Repeated measures analysis of variance was used for inferential statistics. The Kolmogorov-Smirnov test was used to evaluate the normal distribution of the data. Since the data had a normal distribution, Pearson's correlation coefficient was used to investigate the correlation between parameters and the least significant difference (LSD) test was applied to compare the mean values. The level of significance was set at 0.05.

### Ethical Considerations

This study was approved by the Ethics Committee of the Iran University of Medical Sciences (ethics code: IR.IUMS.REC.1398.881). Informed consent was obtained from all participants prior to the study. The data were kept anonymous and confidential. This study was done in accordance with the tenets of the Helsinki Declaration.

##  RESULTS

Forty female nurses participated in this study. Table 1 shows the percentage of subjects with a normal OSDI test (scores of 0–12), mild dry eye (scores of 13–22), moderate dry eye (scores of 23–32), and severe dry eye (scores of 33–100) as well as the subjects with a normal (TBUT > 10 s) and impaired TBUT (TBUT < 10 s) at the beginning of the day shift (8 am), at the end of the day shift (2 pm), at the beginning of the night shift (8 pm), and at the end of the night shift (8 am). According to the OSDI results, 20 subjects (12.5%) were normal, 44 (27.5%) had mild dry eye, 37 (23.1%) had moderate dry eye, and 59 (36.9%) had severe dry eye. According to the TBUT data collected from all four measurements, 42 subjects (26.2%) had normal TBUT results and 118 subjects (73.8%) had dry eye disease.

Table 2 shows the mean TBUT and OSDI values at four measurement times. No significant differences were observed in the TBUT and OSDI values between the beginning of the day shift (8 am) and the beginning of the night shift (8 pm) (*P* > 0.05). The results showed significant differences in both the TBUT and OSDI among other measurement times as well as relative to the beginning of the day and night shifts (*P* < 0.05). According to Table 2, among all OSDI scores obtained from four measurement times, the highest (most severe dry eye) and lowest score (mildest dry eye) were related to the end of the night shift (8 am) and end of the morning shift (2 pm), respectively [Figure 1A]. Similarly, according to the mean TBUT values obtained from all four measurement times, the lowest (most severe dry eye) and highest value (mildest dry eye) were related to the end of the night shift (8 am) and end of the morning shift (2 pm), respectively [Figure 1B].

Table 3 compares the OSDI scores and TBUT values between the beginning and end of the day shift as well as the beginning and end of the night shift. A significant increase was observed in the mean OSDI score at the end of the night shift (8 am) as compared to the beginning of the night shift (8 pm) indicating more dry eye (*P* < 0.05). This is while the OSDI score showed a significant reduction at the end of the day shift (2 pm) as compared to the beginning of the day shift (8 am) indicating less dry eye (*P* < 0.05). Similarly, a significant decrease was found in the mean TBUT value at the end of the night shift (8 am) as compared to the beginning of the night shift (8 pm) indicating more dry eye (*p* < 0.05). This is while the TBUT value showed a significant increase at the end of the day shift (2 pm) as compared to the beginning of the day shift (8 am) indicating less dry eye (*p* < 0.05).

Table 4 presents the correlation between the TBUT value and the OSDI score. According to the results, a significant negative correlation was observed between these two tests in all measurement times (beginning of the day shift: *r* = - 0.478, *P* = 0.002; end of the day shift: *r* = - 0.707, *P* < 0.001; beginning of the night shift: *r* = - 0.556, *P* < 0.001; end of the night shift: *r* = - 0.365, *P* = 0.021).

##  DISCUSSION

In the present study, the lowest rate of dry eye signs and symptoms were related to the end of the day shift (2 pm) among the four measurement times. However, even in this measurement time, 60% of the participants had dry eyes (TBUT < 10 s) according to the TBUT and 70% had mild to severe dry eye according to the OSDI (scores of 13–100). This is while studies have reported a prevalence of 5–30% for dry eye in different age groups.^[12]^ It should be noted that the prevalence of dry eye is 20–73% in high-risk groups.^[13]^

The mean OSDI score is 9.6 (12.2) in healthy normal subjects according to Schiffman et al^[14]^, 7.8 (3.1) according to Miller et al^[15]^, and 3.7 (6.9) according to Tian et al.^[16]^ In our study, the mean OSDI score was higher in nurses with a history of night shift work in all measurements as compared to the above values in normal subjects as well as the score considered normal for the test which was a score of below 12^[10]^ [Table 2]. Moreover, in the present study, the mean TBUT value was below the value considered for normal subjects (more than 10 s)^[11]^ in all measurement times except for the end of the day shift (2 pm) [Table 2]. Therefore, it seems that people with a relatively long history of night shift work report more dry eye signs and symptoms compared to subjects with a negative history.

Regarding the comparison of the mean OSDI scores among four measurement times [Table 2], the results of the present study were consistent with the study by Kawashima et al^[17]^ that found worse dry eye symptoms in people with poor sleep quality. Moreover, the findings of the present study are in line with the results of a study by Makateb et al^[7]^ who found a significant increase in the severity of all four symptoms of the SPEED questionnaire in the morning when they were awake for the whole night compared to the night before. The results of the present study were also consistent with the results of a study conducted by Han et al^[18]^ who found a higher prevalence of dry eye in participants with sleep disorders as compared to subjects without sleep disturbances. However, the results of this study were different from a study conducted by Walker et al^[19]^ who applied the OSDI to subjects with dry eye in the morning and evening shifts and found a lower rate of discomfort in the evening, because in the present study, no significant differences were observed between OSDI scores at the beginning of day and night shifts.

Begley et al^[8]^ evaluated the changes in dry eye symptoms in the morning and evening in patients using the DEQ 2001 questionnaire. Most of the symptoms did not change significantly in the evening as compared to the morning, which was consistent with our results.

Regarding the comparison of the mean TBUT values among the four measurement times [Table 2], the results of the present study were consistent with a study by Lee et al^[6]^ in which the value of TBUT decreased from baseline (before night shift) to 8 am the following day in subjects with night sleep deprivation and recovered at 2 pm. Moreover, the results of the present study were also consistent with a study by Makateb et al^[7]^ who found a significant decrease in the TBUT value at 7 am after the night shift as compared to 7 am before the night shift. Similarly, Walker et al^[19]^ compared the TBUT value between morning and evening in subjects with dry eye and found no significant changes. However, Puinhas et al^[20]^ compared tear film stability between the morning (9–10 am) and evening (5–6 pm) shifts and found a more stable tear film in the morning, which was different from our findings. However, it should be noted that in their study noninvasive tear break-up time (NIBUT) was used to evaluate tear film stability, and the study was conducted on only 10 normal, asymptomatic subjects without night sleep deprivation. Lira et al^[21]^ measured TBUT and NIBUT values in normal subjects without night sleep deprivation in the morning (9–10 am) and evening (5–6 pm) shifts and found lower values in the evening, which were not consistent with the present study.

Results of the current study showed a significant correlation between TBUT and OSDI in four measurement times. This finding was in line with the results of a study by Ozcura et al^[22]^ that reported a significant negative correlation between TBUT and OSDI. Similarly, Begley et al^[8]^ compared dry eye symptoms between dry eye patients and normal subjects in the morning and evening using the DEQ 2001 questionnaire and TBUT and found a significant correlation between these two tests.

The highest rate of symptoms and the lowest TBUT value after the night shift (8 am) may be due to the following reasons: hormonal and neurological changes secondary to night sleep deprivation can change the tear film composition and impair its stability;^[7]^ night sleep deprivation reduces the androgen level and the parasympathetic tone;^[23]^ low levels of androgen can increase tear film evaporation.^[24]^ Tear secretion is influenced by parasympathetic nerves.^[25]^ Some studies have found that disruption of lacrimal innervation results in reduced tear secretion.^[26]^ Therefore, night wakefulness and poor sleep quality may have negative effects on the hormonal profile and parasympathetic tone resulting in dry eye. On the other hand, sleep disturbances are related to ambient light changes that regulate the physiology of human organs through neuronal and hormonal signals.^[2]^ In addition, sleep disturbances are also influenced by changes in the endocrine and autonomic systems and since tear secretion is controlled by these systems, sleep disorders can potentially affect tear quality and quantity.^[7]^ Moreover, according to a study by Nascimento et al, sleep deprivation causes pain hypersensitivity,^[27]^ which may also explain the worsening of dry eye symptoms in the morning following night shift work.

The higher rate of dry eye signs and symptoms at the beginning of the day shift (8 am) compared to the end of the day shift (2 pm), as well as the lower rate of signs and symptoms at the end of the day shift (2 pm) compared to other measurement times, can also be attributed to the direct eyelid pressure on the corneal epithelium during a night's sleep. This reduces tear film stability in the early hours after waking up through the changing of the normal corneal shape, which increases dry eye symptoms. Some contaminants are also collected in the eyes during the night's sleep, which will be gradually removed from the eyes toward noon.^[28,29,30]^

On the other hand, the higher rate of dry eye signs and symptoms at the beginning of the night shift (8 pm) versus the end of the day shift (2 pm) may be due to environmental factors like air conditioning and use of mobile phones and computers during the day, which may decrease the stability of the tear film and increase the symptoms toward the end of the day. However, these changes may also be attributed to inflammatory mediators and tear osmolarity and pH changes toward the end of the day along with tear volume and stability changes.^[29]^ It can also be related to eye fatigue at the end of the day, which may result in more severe signs and symptoms in the beginning, especially at the end of the night shift.^[31]^

In summary, according to the results of the present study, it seems that the prevalence of dry eye signs and symptoms is high among nurses with a relatively long history of night shift work. This study found that the severity of dry eye increased after the night shift with variation over a 24-hr period. Moreover, the results showed a significant correlation between the OSDI and TBUT values at the beginning and at the end of the day and night shifts. Therefore, it is recommended that night shift workers receive regular ophthalmic examinations.

##  Financial Support and Sponsorship

This project was supported by Iran University of Medical Sciences.

## Conflicts of Interest 

None.
